# CHG: A Systematically Integrated Database of Cancer Hallmark Genes

**DOI:** 10.3389/fgene.2020.00029

**Published:** 2020-02-05

**Authors:** Denan Zhang, Diwei Huo, Hongbo Xie, Lingxiang Wu, Juan Zhang, Lei Liu, Qing Jin, Xiujie Chen

**Affiliations:** ^1^ College of Bioinformatics Science and Technology, Harbin Medical University, Harbin, China; ^2^ The 2nd Affiliated Hospital of Harbin Medical University, Harbin, China

**Keywords:** Hallmark genes, mutation, methylation, copy number variation, annotating Hallmark features, database

## Abstract

**Background:**

The analysis of cancer diversity based on a logical framework of hallmarks has greatly improved our understanding of the occurrence, development and metastasis of various cancers.

**Methods:**

We designed Cancer Hallmark Genes (CHG) database which focuses on integrating hallmark genes in a systematic, standard way and annotates the potential roles of the hallmark genes in cancer processes. Following the conceptual criteria description of hallmark function the keywords for each hallmark were manually selected from the literature. Candidate hallmark genes collected were derived from 301 pathways of KEGG database by Lucene and manually corrected.

**Results:**

Based on the variation data, we finally identified the hallmark genes of various types of cancer and constructed CHG. And we also analyzed the relationships among hallmarks and potential characteristics and relationships of hallmark genes based on the topological structures of their networks. We manually confirm the hallmark gene identified by CHG based on literature and database. We also predicted the prognosis of breast cancer, glioblastoma multiforme and kidney papillary cell carcinoma patients based on CHG data.

**Conclusions:**

In summary, CHG, which was constructed based on a hallmark feature set, provides a new perspective for analyzing the diversity and development of cancers.

## Introduction

In 2000, Weinberg et al. (2000) first proposed six hallmarks of cancer, including Sustaining Proliferative Signaling (SPS), Evading Growth Suppressors (EGS), Resisting Cell Death (RCD), Enabling Replicative Immortality (ERI), Inducing Angiogenesis (IA), and Activating Invasion and Metastasis (AIM), which provided a logical framework for conceptualizing a variety of neoplastic diseases. In 2011, they added another four hallmarks to more fully capture the features of cancers, including Genome Instability and Mutation (GIM), Tumor-Promoting Inflammation (TPI), Reprogramming Energy Metabolism (REM), and Evading Immune Destruction (EID) (Hanahan and Weinberg, 2011). The hallmarks of cancer capture the most essential phenotypic characteristics of malignant transformation and progression, but numerous factors involved in this multistep process are still unknown to date. It is undoubtedly that the framework constructed by hallmarks has greatly improved the analysis on diversity of cancers. Balázs Győrffy et al. reviewed the available techniques that are capable of and appropriate for determining the characteristic features of each hallmark (Menyhart et al., 2016). Hallmark capabilities are regulated by partially redundant signaling pathways, and the significance of these pathways depends on the tumor's underlying molecular features. Recently, many studies have focused on the integration of various cancer-related pathways or genes for analysis, and they have found some significant results. In 2011, Jie Li et al. identified high-quality breast cancer prognostic markers and metastasis network modules by integrating hallmark-related genes from GO terms (Li et al., 2010). In 2013, Naif Zaman et al. predicted breast cancer subtype-specific drug targets by exploring the modules (including apoptosis, cell proliferation and cell cycle) in a signaling network assessment of mutations and copy number variations (CNVs) (Zaman et al., 2013). These researches strongly emphasized the importance of constructing gene sets for hallmarks. Moreover, the advantages of the analysis based on a hallmark framework are notable: 1) It reduces feature dimension of cancer (more attention will be focused on the significant genes in each hallmark rather than on all genes, which will reduce the large number of passenger genes analyzed). 2) It is explicable (the results of analysis are depicted more easily). 3) It provides a potential avenue for exploring the mechanism of carcinogenesis. However, the overlap rate of the hallmark genes in current studies is low because the studies use different extraction methods. Furthermore, no gene sets have been systematically collected for the different hallmarks thus far, which makes it difficult to clarify the gene alteration features (including mutations, DNA methylations and CNVs) in each hallmark (Wang et al., 2015).

To address this problem, we established a database called Cancer Hallmark Genes in (CHG), which provides gene sets for the ten hallmarks and the corresponding statistical analysis results, including the frequency of different mutation types (e.g., missense, deletion, insertion), methylation and CNV (e.g., loss or gain) for each gene. To maximize the usage of our database, we collected a total of 22697 samples from TCGA and analyzed the variations of mutation, CNV, and methylation of hallmark genes across 34 cancer types.

Furthermore, we analyzed the relationship among ten hallmarks by Fisher's exact test and unsupervised hierarchical clustering (method 2). Eventually, the hallmarks were clustered into four classes: 1) Reprogramming Energy Metabolism (REM). 2) Activating Invasion and Metastasis (AIM), Evading Growth Suppressors (EGS), Enabling Replicative Immortality (ERI), and Sustaining Proliferative Signaling (SPS). 3) Genome Instability and Mutation (GIM). 4) Tumor-Promoting Inflammation (TPI), Evading Immune Destruction (EID), Resisting Cell Death (RCD), and Inducing Angiogenesis (IA).

Even though the hallmark genes identified in the database came from the confirmed literature and databases, we manually confirmed the top 10 altered (mutation, methylation, CNV) genes of each hallmark to further ensure the accuracy of the data. In addition, we also used several of cancers as examples for further analysis with the CHG data to demonstrate the value of this database at a practical level.

The CHG database is freely available at our website: http://www.bio-bigdata.com/CHG/index.html.

## Materials and Methods

### Data for Hallmarks

In this work, 301 pathways were downloaded from KEGG (version 78.0) (Kanehisa et al., 2017). This data was used for Lucene search and extraction of pathway genes. Gene variant data (7,075 samples of mutation in 34 cancers, 6,177 samples of methylation in 20 cancers, 9445 samples of CNV in 33 cancers) from TCGA (Stratton et al., 2013) were downloaded, where the methylated data was selected as JHU_USC (HumanMethylation 450) and BI (Genome_Wide_SNP_6) was selected for CNV data. These data were used to calculate the frequency of gene variation, and the proportion of different types of variation. The data in this article across DNA methylation, mutation and CNV were from the same samples of TCGA database. In the TCGA database, there are strict rules for the sequencing, processing and analysis, etc. of the samples data and provide standardized data downloading. Human protein-protein interaction data was downloaded from HPRD (Keshava Prasad et al., 2009), STRING (Szklarczyk et al., 2011), BioGRID (Chatraryamontri et al., 2013) and HTRIdb (Bovolenta et al., 2012). Human gene regulation data was downloaded from HTRIdb. These data were used to integrate an integrated gene interaction network. The cDNA data (GRCh38 version and GRCh37 version) was downloaded from Ensembl (Flicek et al., 2014). This data was used for the processing of CNV data (**Supplementary Table 3**).

### The Construction Process of the CHG Database

**Figure 1:**
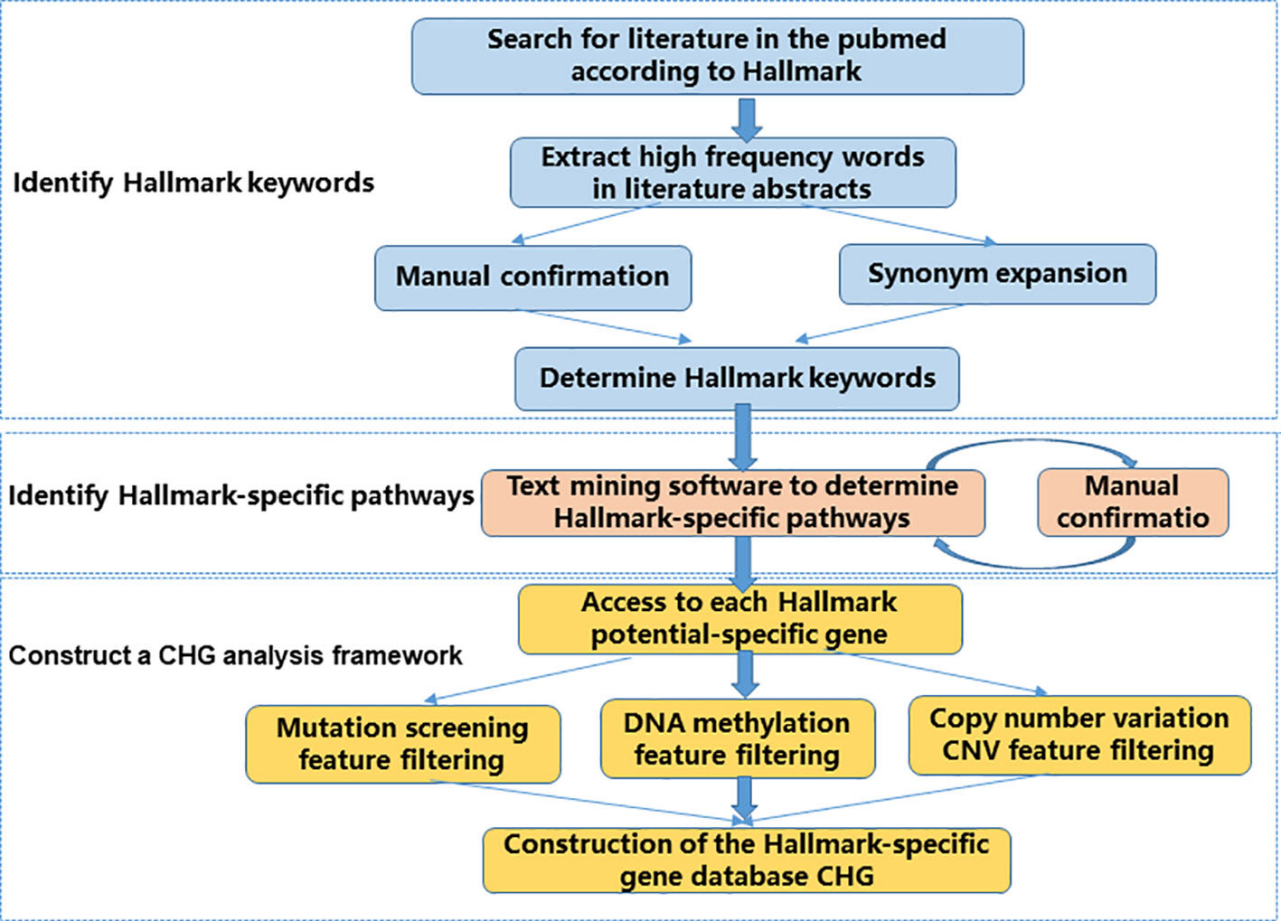
CHG construction flow chart. The CHG database uses a process consisting of three main steps to transform a conceptual description of Hallmark features into real biological processes and their corresponding entities.

Following the conceptual criteria description of hallmark function in the article “Hallmarks of Cancer: The Next Generation,” published in Cell in 2011, we searched the relevant literature in PubMed, and screened the high-frequency descriptive vocabulary appearing in the abstract of the literature as the key words of the corresponding Hallmark. The core idea of our CHG database is to transform the conceptual description of Hallmark features into real biological processes and their corresponding entities. So, we built a process that consists of three main steps (**Figure 1**).

First, we identify the Hallmark description keyword. This step is to materialize the conceptual description of the Hallmark feature. The relevant literature is determined by searching the Hallmark feature description in the literature, and the specific descriptors associated with each Hallmark feature are determined by identifying the high frequency vocabulary in the relevant document abstract. In this step, we manually confirmed the results from the literature scan. In addition to determining that the identified keywords are related to the Hallmark feature, some of the words without more information such as “cancer” and “tumor” are not directly provided to vocabulary. At the same time, we also further enrich the identified Hallmark description keywords through synonym expansion, for example, “apoptosis” and “cell death” (**Supplementary Table 1**).

Second, we use a text mining software package Lucene to identify the Hallmark-specific pathways in the literature and KEGG database based on the Hallmark description keywords identified in the previous step. The result of the identification is manually confirmed again. The manual confirmation step does not add any subjective results, and only in the case of certainty, significant unrelated results due to software recognition errors are removed (**Supplementary Tables 1** , **2**).

Finally, genes with potential specificity in the potential Hallmark-specific pathway were screened from gene mutation level, epigenetic level, and CNV level to construct CHG.

### Cancer Type-Specific Variant Gene

Based on the variation data in TCGA (Montenegro et al., 2015), we calculated the variations of mutation, methylation and CNV for these hallmark genes in different types of cancers. Mutation, CNV, and methylation signatures were used as part of the filtration function in the Hallmark-specific gene screening process in our construction of the CHG database. This is because the relationship between these features and cancer has been confirmed in extensive and in-depth discussions in many previous studies (Kan et al., 2010; Kandoth et al., 2013; Laddha et al., 2014; Wu et al., 2017; Bouras et al., 2019; Sina et al., 2019; Tate et al., 2019). The variations in the characteristics of these different types of cancer not only provide more detailed information for analysis based on the hallmarks but also can be used as a “fingerprint” of cancer type or progression, and this cancer classification can be used as further guidance in prognosis and clinical treatment (**Supplementary Table 3**).

#### Gene Mutation

Based on the somatic mutation (level 2) data for the 34 types of cancers in TCGA, the frequency of each mutated gene was calculated in specific cancers(Chung et al., 2016). To account for the specific action of different somatic mutations in different types or periods of cancers, we mainly studied the following six types of somatic mutations: insertion (INS), deletion (DEL), missense mutations (SNP_mis), nonsense mutations (SNP_non), splice site mutations (SNP_spl), and gene silencing (SNP_sil) (Hu et al., 2018). The proportion of mutation types in each type of cancer was also statistically analyzed (Kan et al., 2010; Kandoth et al., 2013).

#### DNA Methylation

We carried out the following calculations for the level 3 data from 20 human tumors derived from TCGA that simultaneously contained both cancer and control samples (Bouras et al., 2019; Sina et al., 2019):

- Calculate the methylation beta value of each sample (including cancer and normal samples). For genes with multiple methylation sites, the average beta value represents the gene methylation values. The average beta value of the gene in all normal samples was calculated as the methylation level of the control group (Tate et al., 2019);
- When the gene methylation absolute beta value between the cancer and control groups was more than 0.5, it was called a methylation altered gene. We calculated the occurrence frequency of methylation variation and the corresponding beta value of each gene (Tate et al., 2019).
- If the gene's methylated beta value was greater than 0.8 in the cancer samples, it was labeled as H (high), whereas when the methylated beta value was less than 0.2, it was labeled as L (low). We calculated the proportion of genes belonging to H or L (Tate et al., 2019).

#### Copy Number Variation

We analyzed gene segments for the CNV based on level 3 data derived from TCGA and cDNA data from Ensembl in 33 human tumors that simultaneously contained both cancer and control samples. For each pair of samples, if the CNV occurred in only one sample, the default value of the segment in any other sample was 0. Based on experience, we chose 0.2 and -0.2 as the thresholds for altered CNV genes; we marked the gene as a “gain” when the segment value was greater than 0.2 in the cancer samples and as a “loss” when the segment value was less than -0.2 (Laddha et al., 2014). We counted the frequency of CNV in the genes and the proportion of genes belonging to the “gain” and “loss” categories.

### Analysis of Relationships of Hallmarks

We analyzed the relationships among the ten hallmarks by Fisher's exact test and unsupervised hierarchical clustering (Tan et al., 2011; Hashemi et al., 2013). We compared the relationship between the specific gene sets of two hallmarks to the final recognition of the overall relationships among the 10 hallmarks. We separately calculated the number of genes belonging to two hallmarks, only one hallmark and all hallmarks. Based on the null hypothesis of independence between any two hallmarks, we calculated the similarity through Fisher's exact test. Finally, we carried out hierarchical clustering with the 1-P value as the similarity score.

## Results

### The Features of Hallmark Genes Across Cancers

Genome variation is a common phenomenon in cancer, and it is essential to understanding the internal mechanism and prognosis of the tumor in terms of whether the hallmark-related genes have a generally or specifically altered pattern. To this end, we processed the somatic mutation data, methylation data and copy number variant data for 34 cancers in TCGA and analyzed the frequency of somatic mutations, methylation and CNVs in different cancer types (**Table 1**).

**Table 1:** Numbers of pathways and genes of 10 hallmarks.

Hallmarks of cancer	Num. of pathway	Num. of genes
AIM	9	1,101
ERI	4	302
EGS	4	678
RCD	24	1,150
SPS	27	1,263
EID	15	591
TPI	12	619
GIM	10	221
IA	3	483
REM	9	440

**Figure 2:**
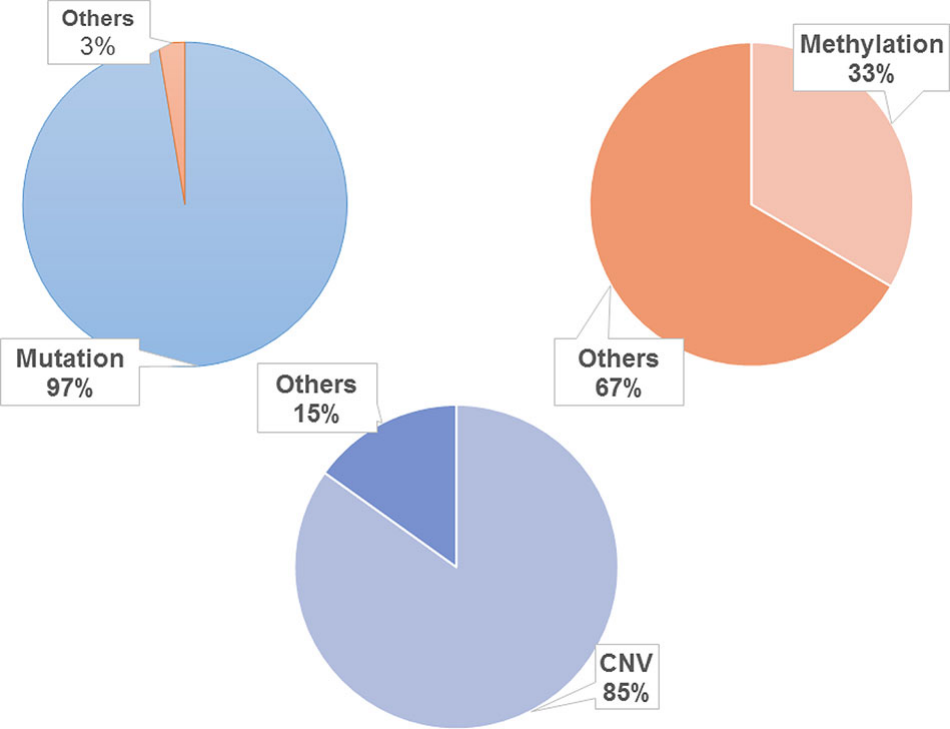
Distribution of genomic changes in 10 hallmarks. The frequency of mutation is about 97.39%, the frequency of methylation is about 33.44% and the frequency of CNV is about 84.88%.

To promote the analysis of carcinogenesis, we mapped the driven mutation, methylation and CNV gene data from TCGA into hallmarks to analyze the altered percentages of all hallmark genes. We found that, among all hallmark genes, 97.39% of the genes were altered by mutation, 33.44% were regulated by methylation, and 84.88% were influenced by CNV (**Figure 2**). In each hallmark, the ratio of genes altered by mutation, methylation and CNV was more than 95% (**Table 2**). These results indicate that the genomic changes in cancer are widespread.

**Table 2:** Ratio of altered Genes in hallmarks.

Hallmarks	Num. of driven Mutation genes	Num. of driven Methylation genes	Num. of driven CNV genes	alteration genes/all driven genes	Ratio of altered Genes
AIM	1,098	334	1,003	1,098/1,101	99.73%
ERI	301	88	277	301/302	99.67%
EGS	617	234	491	645/678	95.13%
RCD	1147	349	1,025	1,147/1,150	99.74%
SPS	1261	356	1,160	1,261/1,263	99.84%
EID	583	258	506	583/591	98.65%
TPI	614	230	537	614/619	99.19%
GIM	220	73	187	220/221	99.55%
IA	482	198	427	482/483	99.79%
REM	438	95	402	438/440	99.55%

For each hallmark, the ratio of genes altered by mutation, methylation, and CNV were more than 95%.

**Figure 3:**
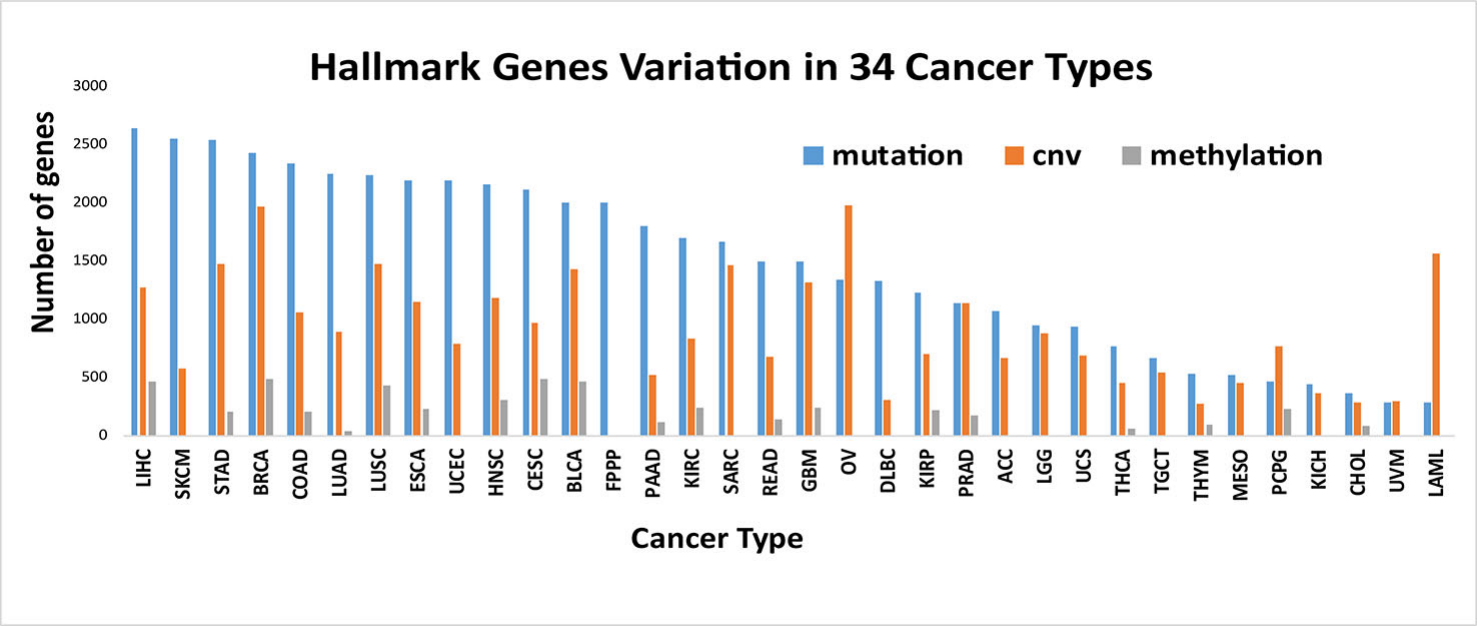
Number of variant genes of Hallmarks in different cancer types. The number of hallmark genes with mutated, differentially methylated and copied in 34 different cancer types. It is showed that different types of cancer have different alteration characteristics.

We counted the number of hallmark genes that are mutated, differentially methylated and copied in 34 different cancer types (**Figure 3**). The results showed that the difference among the number of mutated genes in different cancer types is large, and there is a 9-fold difference between the maximum and the minimum number of mutated genes, with 2644 in LIHC (liver hepatocellular carcinoma) and 281 in LAML (acute myeloid leukemia). The largest number of differentially methylated genes is 490 in BRCA (breast invasive carcinoma), and the smallest number is 34 in LUAD (lung adenocarcinoma). The largest number of differentially CNV genes is 1972 in OV (ovarian serous cystadenocarcinoma), and the smallest number is 267 in THYM (thymoma).

We also found that different types of cancer have different alteration characteristics. As shown in **Figure 3**, some cancers, such as SKCM (skin cutaneous melanoma), ESCA (esophageal carcinoma), LIHC (liver hepatocellular carcinoma), mainly reflect the mutation pattern of the genome, and this is a common pattern in most cancers. Some cancers, such as PCPG (pheochromocytoma and paraganglioma), LAML (acute myeloid leukemia), and OV (ovarian serous cystadenocarcinoma), mainly reflect a pattern of CNV variation, which suggests that we should analyze the specific alteration patterns in specific cancers when uncovering the functional importance of the genomic alterations and the underlying mechanisms that drive cancer development, progression and metastasis in different cancer types.

### Network of Hallmark Genes

**Figure 4:**
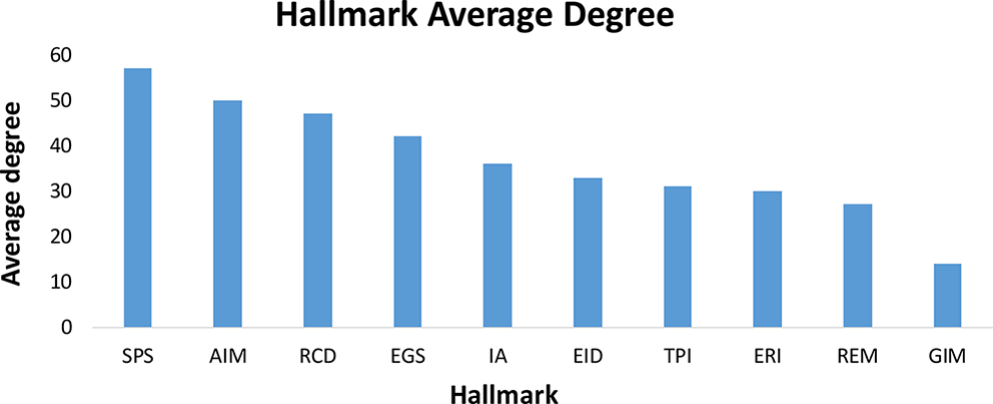
The average degree of ten hallmarks. In addition to the GIM network, the gene interactions inside each hallmark subnetwork were more closely related than the interactions between the 10 hallmark subnetworks.

The potential characteristics and relationships of hallmark genes can be effectively revealed based on the topological structures of their networks. Since the hallmark genes were identified from qualitative analysis without any relevant interaction information, we mapped these hallmark genes onto the integrated protein regulatory network to collect data on the interaction and regulation relationships between the hallmark genes and the extract interactions between the hallmark genes, which resulted in the construction of 10 hallmark subnetworks. The average degree of the integrated protein interactions is 36 and 54 in the regulation network and the entire hallmark network (constructed by all the hallmark interaction genes), respectively. This indicates that the interaction between hallmarks is higher than the average level of integrated protein interactions and shows that hallmark networks are more closely linked. On average, for the 10 hallmark subnetworks, 94% of the hallmark genes were involved in the network (**Supplementary Figure 1**). We performed an analysis of the 10 subnetworks and calculated the degree, betweenness and clustering coefficient of all nodes. We found that, in addition to the GIM network in **Figure 4**, the gene interactions inside each hallmark subnetwork were more closely related than the interactions between the 10 hallmark subnetworks. This result may be due to GIM as the basis of other hallmarks; genetic diversity of GIM will lead to in other hallmark features (Hanahan and Weinberg, 2011). At the same time, we also analyzed the correlation between the degree and number of genes in each subnetwork. The results showed that genes with large degrees often also have larger betweenness, as there was a positive correlation between these variables (**Supplementary Figure 1**).

### Relationship of Hallmarks

**Figure 5:**
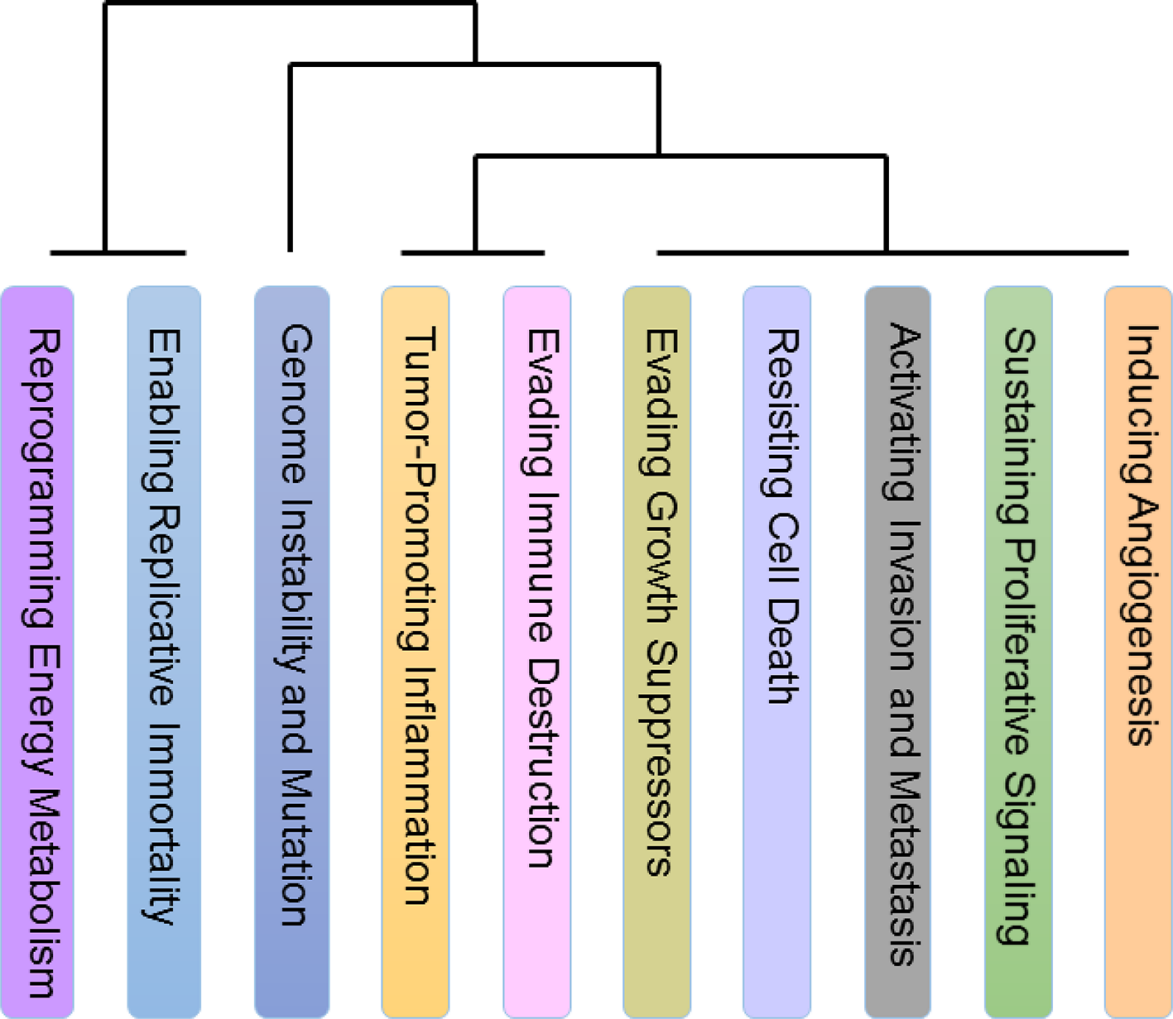
Relationship among ten hallmarks. The relationship among the hallmarks on a pan-cancer scale. There are two classes with only one hallmark, Reprogramming Energy Metabolism (REM) and Genome Instability and Mutation (GIM) and both of these hallmarks are clearly different from the other hallmarks in terms of their mechanisms. In addition, the similarity among the hallmarks Activating Invasion and Metastasis (AIM), Evading Growth Suppressors (EGS), Enabling Replicative Immortality (ERI), and Sustaining Proliferative Signaling (SPS) is prominent. Many of the hallmarks in this set are related to the preliminary stage of cancers. The last class includes Tumor-Promoting Inflammation (TPI), Evading Immune Destruction (EID), Resisting Cell Death (RCD), and Inducing Angiogenesis (IA). Noticeably, tumor-promoting inflammation may activate the response of immune system, and many recent studies have focused on the relationship between inflammation and the immune system in cancers.

Ten types of hallmarks described different aspects of the tumor characteristics, but there were few relationships mentioned between these characteristics on a pan-cancer scale. To this end, we analyzed the relationship among the hallmarks and divided the ten hallmarks into four classes (**Figure 5**). Interestingly, we found two classes with only one hallmark, namely, *Reprogramming Energy Metabolism (REM)* and *Genome Instability and Mutation (GIM)*. This result is reasonable, as both of these hallmarks are clearly different from the other hallmarks in terms of their mechanisms. As we know, almost all types of cancers are caused by DNA mutation or genome structure alterations and are followed by the appearance of other hallmarks.

In addition, the similarity among the hallmarks Activating Invasion and Metastasis (AIM), Evading Growth Suppressors (EGS), Enabling Replicative Immortality (ERI) and Sustaining Proliferative Signaling (SPS) is prominent. Many of the hallmarks in this set are related to the preliminary stage of cancers (Hanahan and Weinberg, 2000; Hanahan and Weinberg, 2011). One confusing inclusion in the set is AIM, which is a hallmark that is considered to be related to the end stage of cancers. However, recent research has also found that AIM occurs in early cancers as well (Hanahan and Weinberg, 2011).

The last class includes *Tumor-Promoting Inflammation (TPI), Evading Immune Destruction (EID), Resisting Cell Death (RCD)*, and *Inducing Angiogenesis (IA)*. Noticeably, tumor-promoting inflammation may activate the response of immune system, and many recent studies have focused on the relationship between inflammation and the immune system in cancers (Grivennikov et al., 2010; Tan et al., 2011; Elinav et al., 2013; Hashemi et al., 2013).

**Figure 6:**
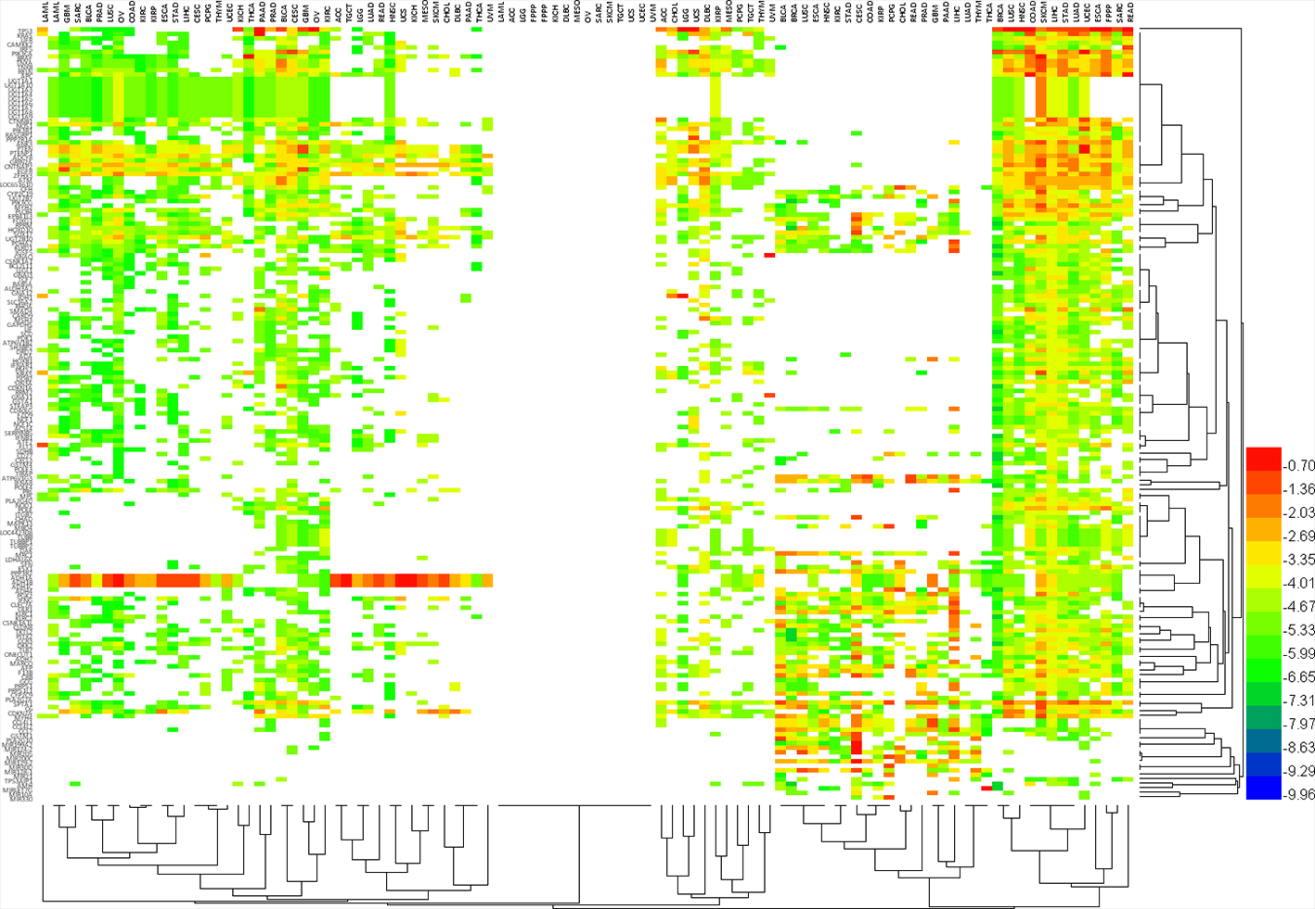
The pattern of characteristic variation of Hallmark genes in 34 different cancers. Heat map shows major differences between the altered features (e.g., mutation, CNV or methylation) of each hallmark gene as altered patterns in different types of cancer. In fact, these features can be used as simple markers for distinguishing cancer types.

In addition, we further analyzed the patterns of characteristic variation of the hallmark genes (**Figure 6**) in 34 different cancers (**Supplementary Table 3**). We looked at the top 10 altered features (e.g., mutation, CNV or methylation) of each hallmark gene as the Typical Characteristics of the Hallmark Gene (TCHG, **Supplementary Table 4**). In heat map analysis, we can clearly find major differences between the TCHGs as altered patterns in different types of cancer. In fact, these features can be used as simple markers for distinguishing cancer types.

### Validation of CHG Data

Although the hallmark-related genes identified in the database came from the confirmed literature and databases, we manually confirmed the TCHG to further ensure the accuracy of the data. Considering the very large dataset that we had to confirm, we have currently verified only the top 10 altered (mutation, methylation, CNV) genes of each hallmark. Over 92% of the typical characteristic genes have explanations of their specific hallmark functions in the literature, which demonstrates the accuracy and precision of the CHG data on a theoretical level (**Supplementary Table 4**).

In addition, we compared the results of this study with existing Sanger Cancer Gene Census databases (Futreal et al., 2004). The Sanger Cancer Gene Census database not only describes the genomic features of cancer-related genes themselves, but also includes information on tissue distribution, mutation information and protein structure. We also compared 699 cancer-related genes identified in the Sanger Cancer Gene Census database with the Typical Characteristics of the Hallmark Gene (TCHG) we identified. Of the 139 Hallmark-related TCHG genes we identified, 69 were also included in the Sanger database, accounting for 49.7%. These results also confirm the accuracy of our results. For other genes that are not included in the Sanger database, we also confirm their important role in cancer-related biological processes through literature verification, such as ETS1 (Watabe et al., 1998; Fujimoto et al., 2004; Zhang et al., 2014; Li et al., 2015) and RHOA (Lee et al., 2015; Zeng et al., 2015; Sun et al., 2016) in hallmark “Activating Invasion and Metastasis”.

### CHG Case Study

In addition, we used breast cancer data that was labeled as recurrent or not recurrent as samples for further analysis based on the CHG data. These analyses can be used as an example of the applications of the CHG database and can also prove the value of this database at a practical level. We performed a significant enrichment analysis of the differentially expressed genes based on data from 159 breast cancer patients from GEO with a significance level of p < 0.01. The sample group and the control group were patient data with and without recurrence, respectively. In particular, these differentially expressed genes were filtered by hallmark genes from the CHG database before performing the enrichment analysis. We found that these genes were enriched in 2 out of the 10 hallmarks, corresponding to the hallmarks whose main functions include *Genome Instability and Mutation (GIM)* and *Tumor-Promoting Inflammation (TPI)* (**Table 3**). It is well known that tumor development is jointly promoted by cell-intrinsic and cell-extrinsic factors. The hallmarks in **Table 3** include risk factors for tumor recurrence that are both extracellular (*Tumor-Promoting Inflammation*) and intracellular (*Genome Instability and Mutation*). These results not only expressed the theoretical interpretation of the enrichment analysis but also reflected the significance of the hallmark genes in the CHG database.

**Table 3:** Hallmark function of differentially expressed genes based on 137 breast cancer data.

Hallmark	P-value
Genome Instability and Mutation	0.000121
Tumor-Promoting Inflammation	0.004591

**Figure 7:**
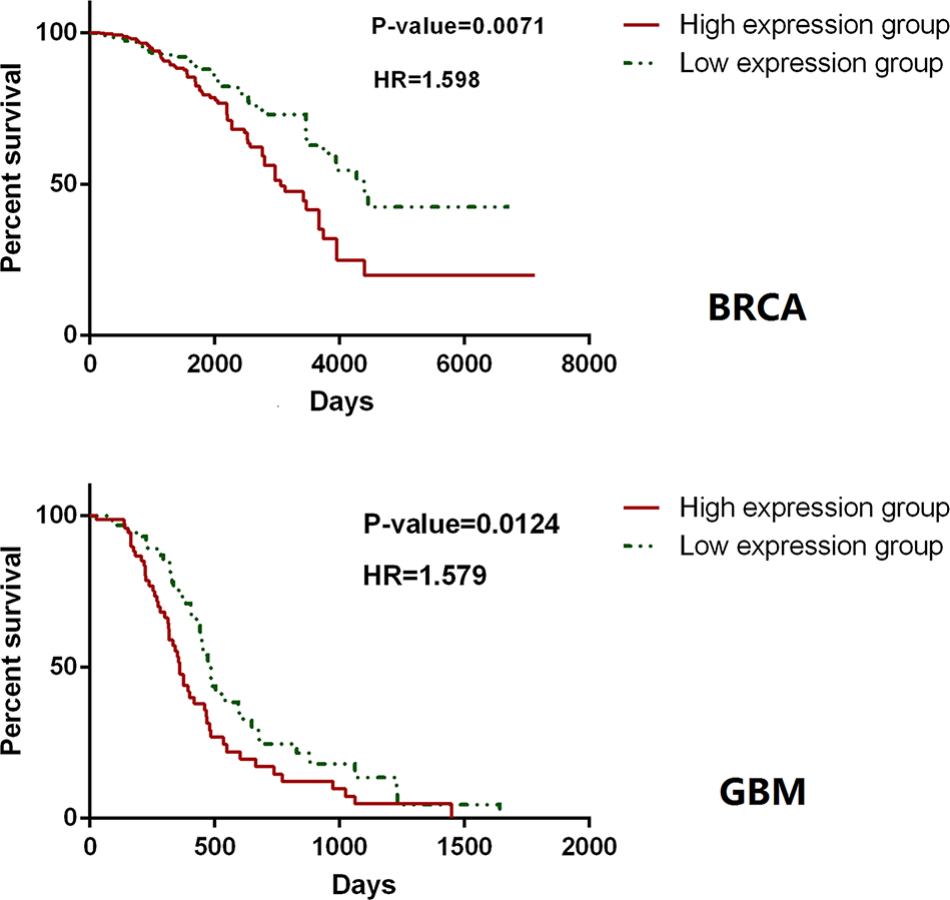
Hallmark genes could clearly distinguish the length of the survival time in the prognosis. In a survival analysis of 1,183 breast cancer patients (up) and 156 glioblastoma multiforme patients (down), only the expression level of hallmark genes could clearly distinguish the length of the survival time in the prognosis.

**Figure 8:**
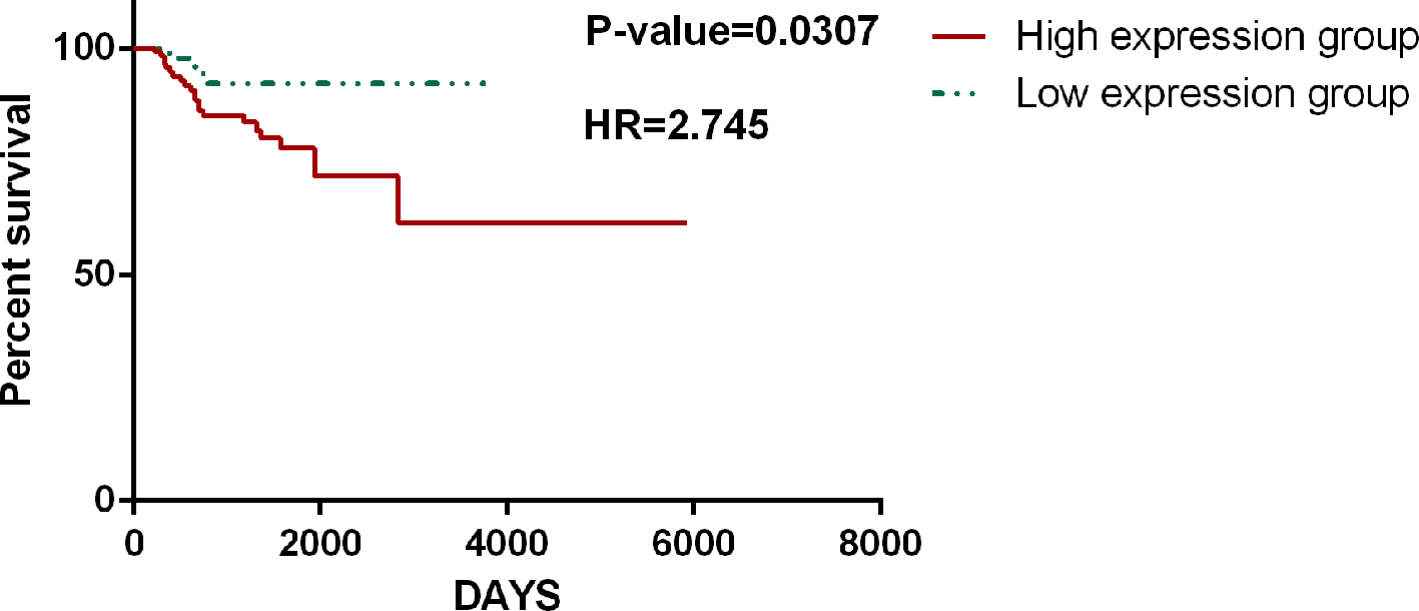
CHG hallmark genes can be used as a marker to determine the recurrence of cancer. An analysis of the survival data of 284 KIRP (kidney papillary cell carcinoma) patients with 27 recurrence cases shows that the hallmark genes identified in CHG have good sensitivity for distinguishing cancer recurrence.

The accuracy and specificity of the hallmark genes identified in CHG can also be confirmed by our analysis of the survival data for cancer patients. The survival analysis based on TCGA data was carried out with only hallmark genes as a single block, and it showed that patient groups with differentially expressed (compared to the average expression level) hallmark markers could clearly distinguish the prognosis of patients with high statistical significance. Similar results have been found in many types of cancer. For instance, in a survival analysis of 1183 breast cancer patients and 156 glioblastoma multiforme patients, only the expression level of hallmark genes could clearly distinguish the length of the survival time in the prognosis (**Figure 7**). In addition, the hallmark gene identified by CHG can also be used as a marker to determine the recurrence of cancer to some extent. An analysis of the survival data of 284 KIRP (kidney papillary cell carcinoma) patients with 27 recurrence cases in **Figure 8** shows that the hallmark genes identified in CHG have good sensitivity for distinguishing cancer recurrence. These results fully showed that the variation characteristics of the hallmark-related genes in CHG were representative, and they could be directly applied to rapid qualitative analysis.

## Discussion

Since Weinberg et al. firstly established the hallmarks for cancer in 2000, many studies have focused on the analysis of cancer based on a framework constructed by these hallmarks. In addition, in 2011, the number of hallmarks increased to ten, which indicates that the features of cancer may be exceedingly complex. Perhaps unsurprisingly, in 2013, another hallmark, *Aberrant Alternative Splicing*, was proposed by Michael Ladomery (Ladomery, 2013). It has been reported that the vast majority of human genes, possibly over 94%, are alternatively spliced (Pan et al., 2008). In 2015, MF Montenegro et al. targeted the epigenetic machinery of cancer cells and noted that there was increasing evidence linking the aberrant regulation of methylation to carcinogenesis (Montenegro et al., 2015), which implied that it may be a potential hallmark for cancer. In 2015, Mamatha Bhat et al. published a review about the translation machinery in cancer. They mentioned that translation played a major role in the regulation of gene expression, and the dysregulation of this process is considered a hallmark of cancer.

The CHG database that we constructed is based on the ten hallmarks that Weinberg proposed in 2011. As a specifically designed framework constructed from a hallmark database, CHG can provide a new perspective for an analysis of the diversity and development of cancers as well as a convenient method for in-depth data mining. The CHG database focused on integrating hallmark genes, annotating the potential roles of hallmark features in human cancer processes, and evaluating the relationships of the ten hallmarks by constructing hallmark networks and calculating the degree and distance between genes belonging to each network. Even though the hallmark-related genes identified in the database have been confirmed by consensus from the literature and databases, we manually confirmed the top 10 altered (mutation, methylation, CNV) genes in each hallmark to further ensure the accuracy of our data.

According to our plan, CHG database will be updated regularly every year to supplement the new findings in hallmark field or revise the existing results. We will also follow up the study of cancer hallmarks, the update of important data source (such as revision of TCGA or KEGG) and improve the practicality of CHG database in mechanism interpretation and clinical aspects. All of old version database would also be maintained and access to downloaded. The difference of each version of database would be listed.

Furthermore, over the past decade, analysis based on the integration of multiple datasets has become quite prevalent. In 2013, Du et al. (Du et al., 2013) analyzed clinically relevant long noncoding RNAs in human cancer by integrating SCNA (somatic copy number alteration), lncRNA and clinical data. In 2014, Wu et al., (2014) predicted disease-causing nonsynonymous single nucleotide variants by integrating multiple genomic datasets. Sanchez et al., (2014) integrated an analysis of Chip-Seq and RNA-Seq data to unveil an lncRNA tumor suppressor signature. Many studies, such as the work of Peng et al., have determined that miRNAs are a widely regulated regulatory mechanism in cancer (Peng et al., 2019b). Hence, it is worthwhile to integrate non-coding RNA (including miRNA, lncRNA, etc.) (Cheng et al., 2016; Cheng et al., 2019), fusion genes and drug information into a database. We have set out to construct a network that is comprised of these non-coding RNAs, genes and drugs. We hope that the next step will be to provide an online analysis tool (such as Peng et al., 2019a; Peng et al., 2019c) to provide further personalized analysis. We will gather these resources into the database in the next version, and we anticipate that the database will help promote the analysis of cancer and the identification of valuable drug targets.

## Data Availability Statement

The CHG database is freely available at our website: http://www.bio-bigdata.com/CHG/index.html.

## Author Contributions

DZ, DH, HX, and LW contributed equally to this work and should be considered co-first authors. JZ, LL, and HX collected data and conducts calculation and analysis. DH, QJ, and XC analyzed the results. DZ and LW wrote the paper. All authors reviewed the manuscript.

## Funding

This work was supported by the National Natural Science Foundation of China [Grant No. 61671191, 61971166, 61701142, 61802090].

## Conflict of Interest

The authors declare that the research was conducted in the absence of any commercial or financial relationships that could be construed as a potential conflict of interest.

## Supplementary Material

Supplementary Figure 1 Topological characteristics of hallmark gene networks.

Supplementary Table 1 Characteristic pathways of different hallmarker with mapping keys.

Supplementary Table 2 Genesets of 10 hallmarker.

Supplementary Table 3 Specific genes in 34 cancer type of mutation.

Supplementary Table 4 Literature validation of TOP10 altered (mutation, methylation, CNV) genes (TCHGs) of each hallmark.
