# Supplementary figures and images for: CHG: A Systematically Integrated Database of Cancer Hallmark Genes
Source: Front Genet. 2020 Feb 5;11:29. doi: 10.3389/fgene.2020.00029 (PMC7013921; doi:10.3389/fgene.2020.00029)

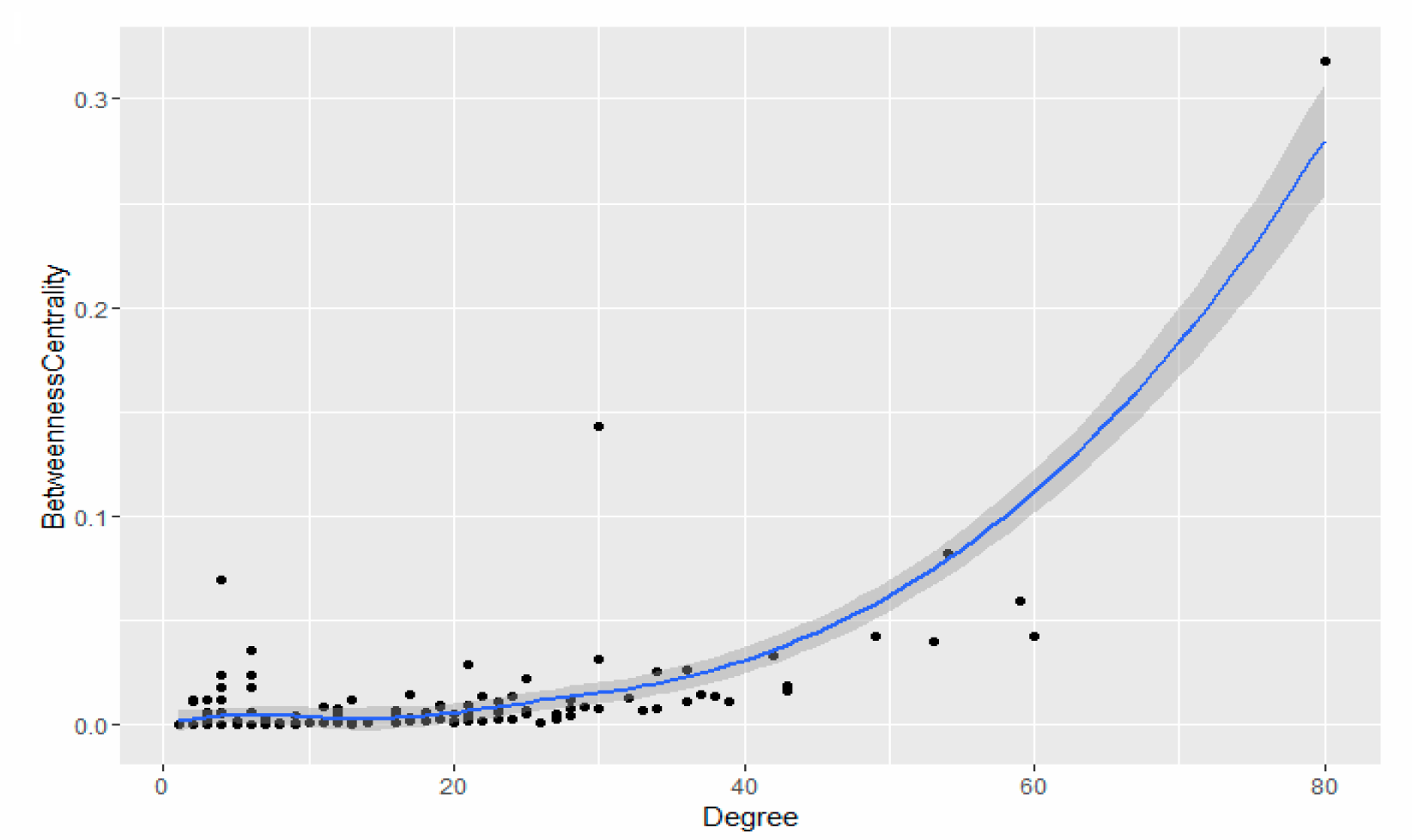

Supplement: Supplementary Figure 1 — Topological characteristics of hallmark gene networks. [file Image_1.tif]
